# Supplementary material for: Fecal Microbiota Dynamics Reveal the Feasibility of Early Weaning of Yak Calves under Conventional Grazing System
Source: Biology (Basel). 2021 Dec 26;11(1):31. doi: 10.3390/biology11010031 (PMC8773362; doi:10.3390/biology11010031)
Supplement: Supplementary file 1 [file biology-11-00031-s001.zip › biology-1493198-supplementary.pdf]

Supplementary Material

Table Legends

Table S1. Comparison of alpha diversity of fecal microbiota in yak and cattle calves at different months after weaning

Breed group	VS. Group	Chao1 species index			Shannon diversity index		
		W	p.value	q.value	H	p.value	q.value
Yak calves	YW1M vs. YW2M	2.482	0.115	0.293	3.982	0.046	0.092
	YW1M vs. YW5M	1.591	0.207	0.387	3.188	0.074	0.130
	YW1M vs. YW8M	0.276	0.600	0.730	3.574	0.059	0.110
	YW2M vs. YW5M	0.622	0.430	0.603	0.176	0.674	0.821
	YW2M vs. YW8M	1.864	0.172	0.344	0.044	0.834	0.865
	YW5M vs. YW8M	1.337	0.248	0.433	0.176	0.674	0.821
Cattle calves	CW1M vs. CW2M	0.000	1.000	1.000	2.824	0.093	0.144
	CW1M vs. CW5M	8.660	0.003	0.046	11.294	0.001	0.018
	CW1M vs. CW8M	2.175	0.140	0.304	9.926	0.002	0.018
	CW2M vs. CW5M	9.276	0.002	0.046	8.647	0.003	0.018
	CW2M vs. CW8M	3.574	0.059	0.205	4.412	0.036	0.083
	CW5M vs. CW8M	3.188	0.074	0.208	5.835	0.016	0.055
Yak calves vs. Cattle calves	CW1M vs. YW1M	0.177	0.674	0.755	0.099	0.753	0.843
	CW2M vs. YW2M	6.893	0.009	0.081	3.982	0.046	0.092
	CW5M vs. YW5M	2.165	0.141	0.304	4.864	0.027	0.077
	CW8M vs. YW8M	0.621	0.431	0.603	0.000	1.000	1.000

Note: W: Wilcox test statistics; p.value: Willcox test, less than 0.05 indicates significant differences between groups; q.value: Benjamini-Hochberg FDR correction were used in two-group analysis.

Table S2. The comparison of the fecal microbial functions of yak and cattle calves after weaning based on KEGG level 2 annotation

Samples type	Vs. group	KEGG level 2 annotation	Group1		Group2		p.value	q.value
			Avg	sd	Avg	sd		
Yak Calves	YW1M vs YW2M	Viral protein families	-2.9567	0.1837	-2.6300	0.1494	0.0047	0.0467
		Substance dependence	-1.7944	0.1026	-2.1560	0.2937	0.0148	0.0758
		Sensory system	-3.2271	0.9055	-4.5537	1.2959	0.0281	0.1125
		Excretory system	-1.1869	0.0845	-1.1010	0.0418	0.0379	0.1356
	YW2M vs YW5M	Neurodegenerative disease	-0.0107	0.0367	0.0336	0.0238	0.0070	0.0546
		Infectious disease: viral	-0.6201	0.0264	-0.5491	0.0751	0.0148	0.0758
	YW5M vs YW8M	Neurodegenerative disease	0.0336	0.0238	0.0176	0.0032	0.0002	0.0115
Cattle Calves	CW2M vs CW5M	Viral protein families	-2.7857	0.0998	-2.4326	0.1354	0.0002	0.0115
		Substance dependence	-2.0358	0.2474	-2.4395	0.0656	0.0003	0.0136
		Sensory system	-3.9819	1.2594	-5.5176	0.0466	0.0019	0.0304
		Infectious disease: parasitic	-1.1987	0.0309	-1.1371	0.0385	0.0070	0.0546
		Cellular community - eukaryotes	-3.3512	0.3378	-2.9952	0.2426	0.0207	0.0932
	CW5M vs CW8M	Xenobiotics biodegradation and metabolism	0.4132	0.0259	0.4445	0.0113	0.0070	0.0546
		Viral protein families	-2.4326	0.1354	-2.7840	0.1573	0.0006	0.0162
		Unclassified: metabolism	1.0070	0.0265	1.0351	0.0098	0.0379	0.1356
		Transport and catabolism	0.1015	0.0402	0.1562	0.0177	0.0030	0.0384
		Translation	1.1261	0.0178	1.1466	0.0108	0.0207	0.0932
		Transcription	-0.2065	0.0153	-0.1900	0.0092	0.0379	0.1356
		Signal transduction	0.8776	0.0297	0.9083	0.0130	0.0499	0.1606
		Sensory system	-5.5176	0.0466	-5.4556	0.0444	0.0104	0.0594
		Replication and repair	1.0715	0.0206	1.0955	0.0111	0.0148	0.0758
		Protein families: signaling and cellular processes	1.6520	0.0287	1.6815	0.0143	0.0379	0.1356
		Protein families: metabolism	1.3818	0.0217	1.4053	0.0111	0.0281	0.1125
		Protein families: genetic information processing	1.8489	0.0220	1.8737	0.0117	0.0148	0.0758
		Poorly characterized	0.8709	0.0293	0.9010	0.0100	0.0379	0.1356
		Nucleotide metabolism	1.0354	0.0220	1.0610	0.0103	0.0148	0.0758
		Nervous system	-0.1470	0.0257	-0.1124	0.0112	0.0019	0.0304
		Metabolism of terpenoids and polyketides	0.5493	0.0172	0.5673	0.0108	0.0281	0.1125
		Metabolism of other amino acids	0.6852	0.0240	0.7137	0.0103	0.0104	0.0594
		Metabolism of cofactors and vitamins	1.1986	0.0236	1.2269	0.0108	0.0104	0.0594
		Lipid metabolism	0.8260	0.0249	0.8527	0.0117	0.0379	0.1356
		Infectious disease: parasitic	-1.1371	0.0385	-1.1767	0.0301	0.0499	0.1606
		Infectious disease: bacterial	0.4128	0.0248	0.4391	0.0119	0.0379	0.1356
		Immune system	0.0332	0.0234	0.0566	0.0138	0.0379	0.1356
		Glycan biosynthesis and metabolism	0.9416	0.0272	0.9774	0.0102	0.0047	0.0467
		Folding, sorting and degradation	0.7659	0.0222	0.7921	0.0105	0.0148	0.0758
		Excretory system	-1.1342	0.0534	-1.0755	0.0183	0.0070	0.0546
		Environmental adaptation	-0.0140	0.0252	0.0142	0.0111	0.0148	0.0758
		Energy metabolism	1.2081	0.0230	1.2345	0.0095	0.0148	0.0758

		Endocrine system	0.4625	0.0218	0.4844	0.0106	0.0379	0.1356
		Endocrine and metabolic disease	-0.0608	0.0183	-0.0418	0.0115	0.0207	0.0932
		Drug resistance: antineoplastic	-0.1228	0.0233	-0.0921	0.0104	0.0047	0.0467
		Drug resistance: antimicrobial	0.5756	0.0360	0.6200	0.0116	0.0047	0.0467
		Development and regeneration	-1.1888	0.0537	-1.1121	0.0167	0.0019	0.0304
		Cellular community - prokaryotes	0.8313	0.0272	0.8612	0.0157	0.0207	0.0932
		Cell growth and death	0.4872	0.0207	0.5130	0.0098	0.0070	0.0546
		Cardiovascular disease	0.1863	0.0205	0.2090	0.0104	0.0281	0.1125
		Carbohydrate metabolism	1.5510	0.0259	1.5813	0.0098	0.0104	0.0594
		Cancer: overview	0.3214	0.0243	0.3501	0.0097	0.0047	0.0467
		Biosynthesis of other secondary metabolites	0.7996	0.0227	0.8268	0.0101	0.0104	0.0594
		Amino acid metabolism	1.4234	0.0230	1.4494	0.0110	0.0207	0.0932
		Aging	0.0617	0.0252	0.0914	0.0113	0.0148	0.0758
Yak Calves Vs. Cattle Calves	CW1M vs YW1M	Transport and catabolism	0.1475	0.0548	0.0869	0.0636	0.0379	0.1356
		Substance dependence	-2.1492	0.4981	-1.7944	0.1026	0.0207	0.0932
		Nervous system	-0.1461	0.0299	-0.1718	0.0389	0.0499	0.1606
		Metabolism of other amino acids	0.6737	0.0263	0.6511	0.0366	0.0499	0.1606
		Endocrine system	0.4341	0.0257	0.4075	0.0374	0.0281	0.1125
		Drug resistance: antineoplastic	-0.1385	0.0260	-0.1589	0.0443	0.0499	0.1606
		Development and regeneration	-1.1191	0.0673	-1.2254	0.0517	0.0019	0.0304
		Carbohydrate metabolism	1.5420	0.0243	1.5174	0.0445	0.0499	0.1606
		Cancer: overview	0.3055	0.0228	0.2744	0.0237	0.0070	0.0546
	CW2M vs YW2M	Viral protein families	-2.7857	0.0998	-2.6300	0.1494	0.0281	0.1125
		Cellular community - eukaryotes	-3.3512	0.3378	-3.0540	0.2278	0.0499	0.1606
	CW5M vs YW5M	Xenobiotics biodegradation and metabolism	0.4132	0.0259	0.4454	0.0295	0.0499	0.1606
		Viral protein families	-2.4326	0.1354	-2.7020	0.1681	0.0019	0.0304
		Signaling molecules and interaction	-4.3766	0.9923	-5.3784	0.3486	0.0047	0.0467
		Neurodegenerative disease	0.0029	0.0208	0.0336	0.0238	0.0070	0.0546
		Immune disease	-0.8712	0.0118	-0.8720	0.0554	0.0499	0.1606
		Development and regeneration	-1.1888	0.0537	-1.1375	0.0423	0.0379	0.1356
		Circulatory system	-3.0486	0.1221	-2.7502	0.5649	0.0499	0.1606
	CW8M vs YW8M	Infectious disease: viral	-0.6134	0.0365	-0.5502	0.0640	0.0499	0.1606
		Infectious disease: parasitic	-1.1767	0.0301	-1.1411	0.0403	0.0499	0.1606
		Development and regeneration	-1.1121	0.0167	-1.1363	0.0278	0.0499	0.1606
		Cellular community - eukaryotes	-3.0899	0.3243	-2.7569	0.2750	0.0379	0.1356

Note: Avg: Average value; sd: Standard deviation; p.value: Wilcox test P value, less than 0.05 indicates significant differences between groups; q.value: Benjamini-Hochberg FDR correction were used in two-group analysis.

Figure Legends

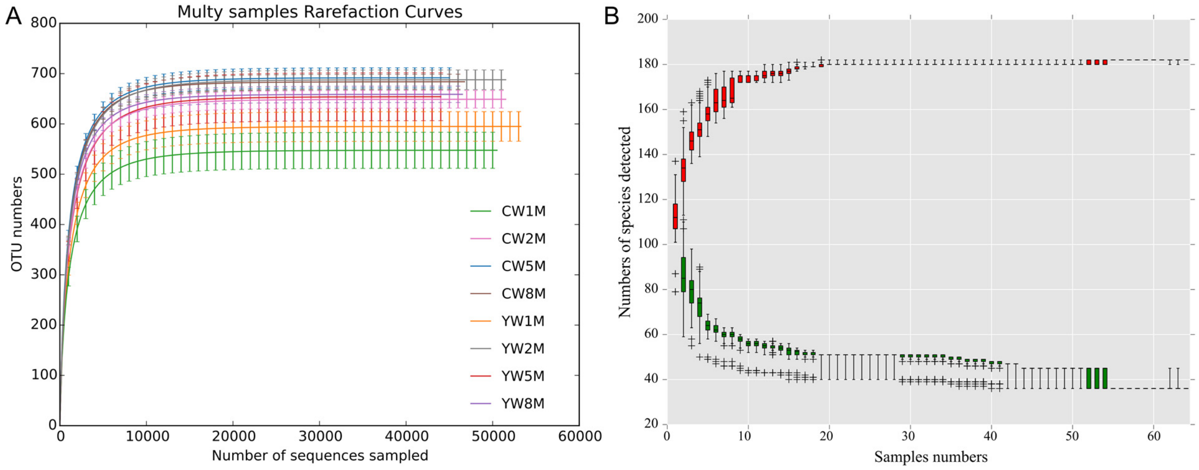

Figure S1. Diversity analysis of fecal microbial community of yak and cattle calves in different months after weaning. (**A**) Rarefaction curve based on the observed ASVs for each sample. (**B**) Species accumulation boxplot for each sample based on the genus level ASVs for each sample. A single red box reflects the total number of species in the sample, and the total red box forms a cumulative curve, reflecting the rate of new species emergence under continuous sampling; a single green box reflects the number of species shared in the sample.

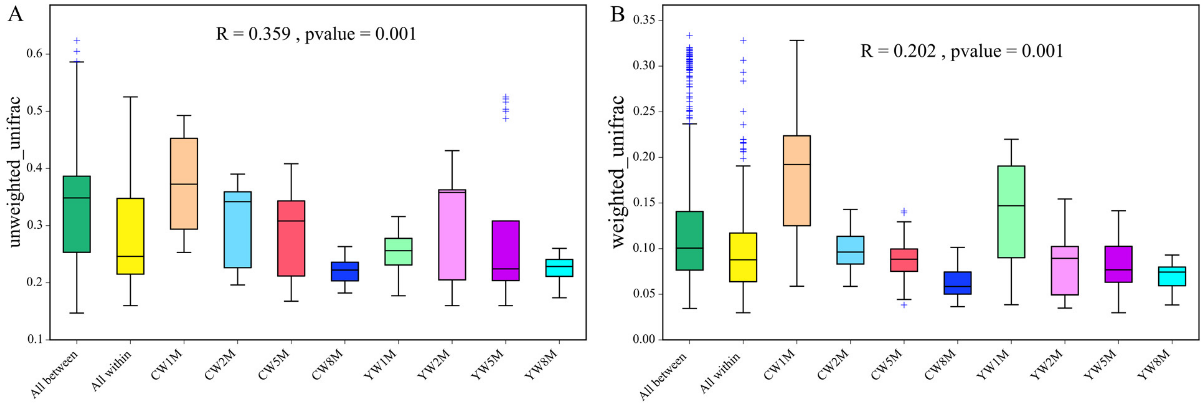

Figure S2. Beta diversity analysis of fecal microbial community in yak and cattle calves at different months after weaning. (**A**) Unweighted UniFrac distance boxplots showing differences between weaned yak and cattle calves and within groups by ANOSIM. (**A**) Weighted UniFrac distance boxplots showing differences between weaned yak and cattle calves and within groups by ANOSIM.

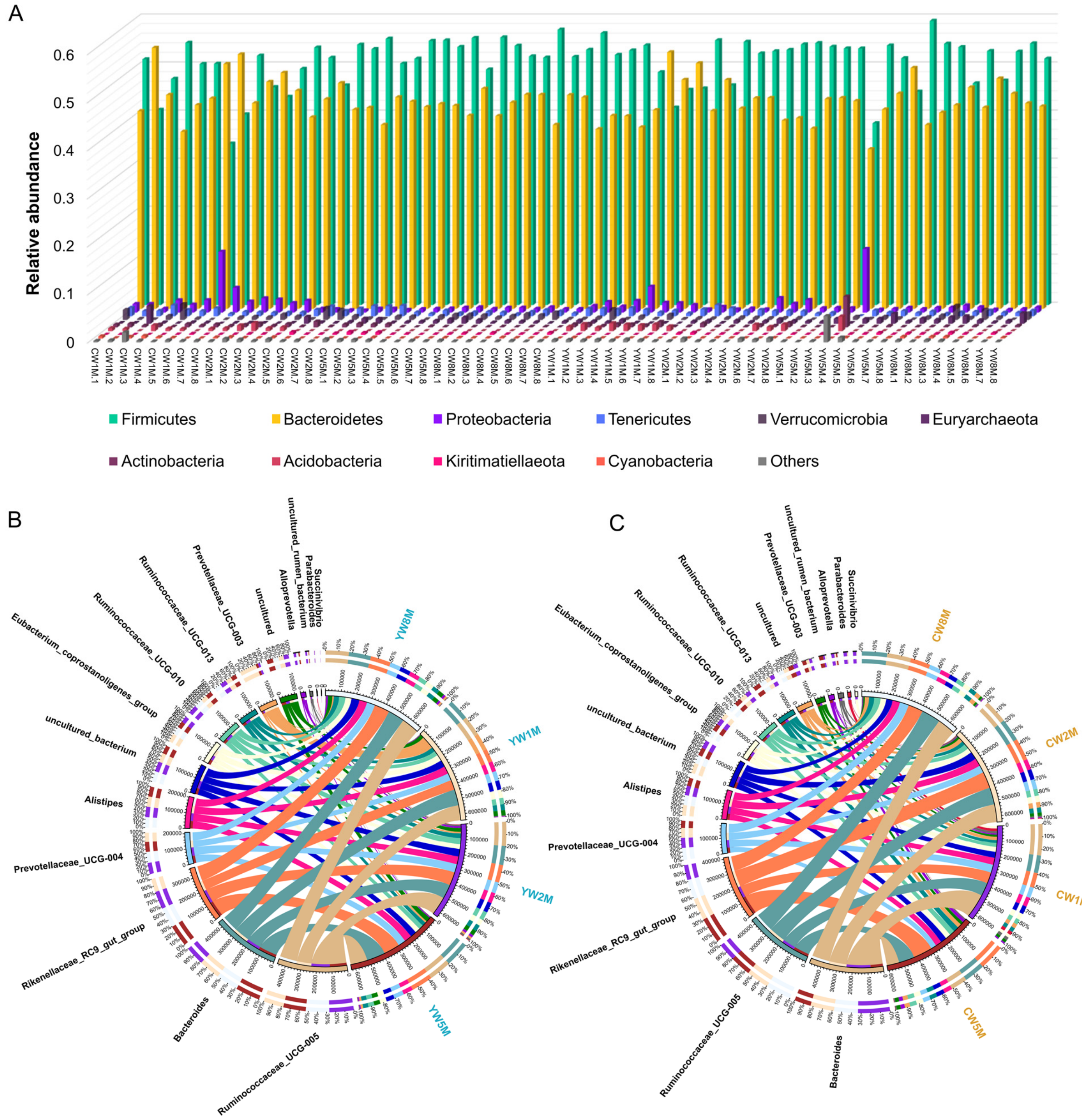

Figure S3. The composition of fecal microbial community in yak and cattle calves in different months after weaning. (**A**) Bar plot shows the composition of fecal microbial communities at the phylum level (Top10) of yak and cattle calves in different months after weaning. Circos diagram shows the composition of fecal microbial communities at the genus level (Top15) of yak (**B**) and cattle calves (**C**) in different months after weaning, respectively. The length of the bars on the outer ring and the numbers on the inner ring represent the percentage of relative abundance of genera detected in each sample and the number of sequences in each sample, respectively. The bands with different colors show the source of each sequence affiliated with different clusters.

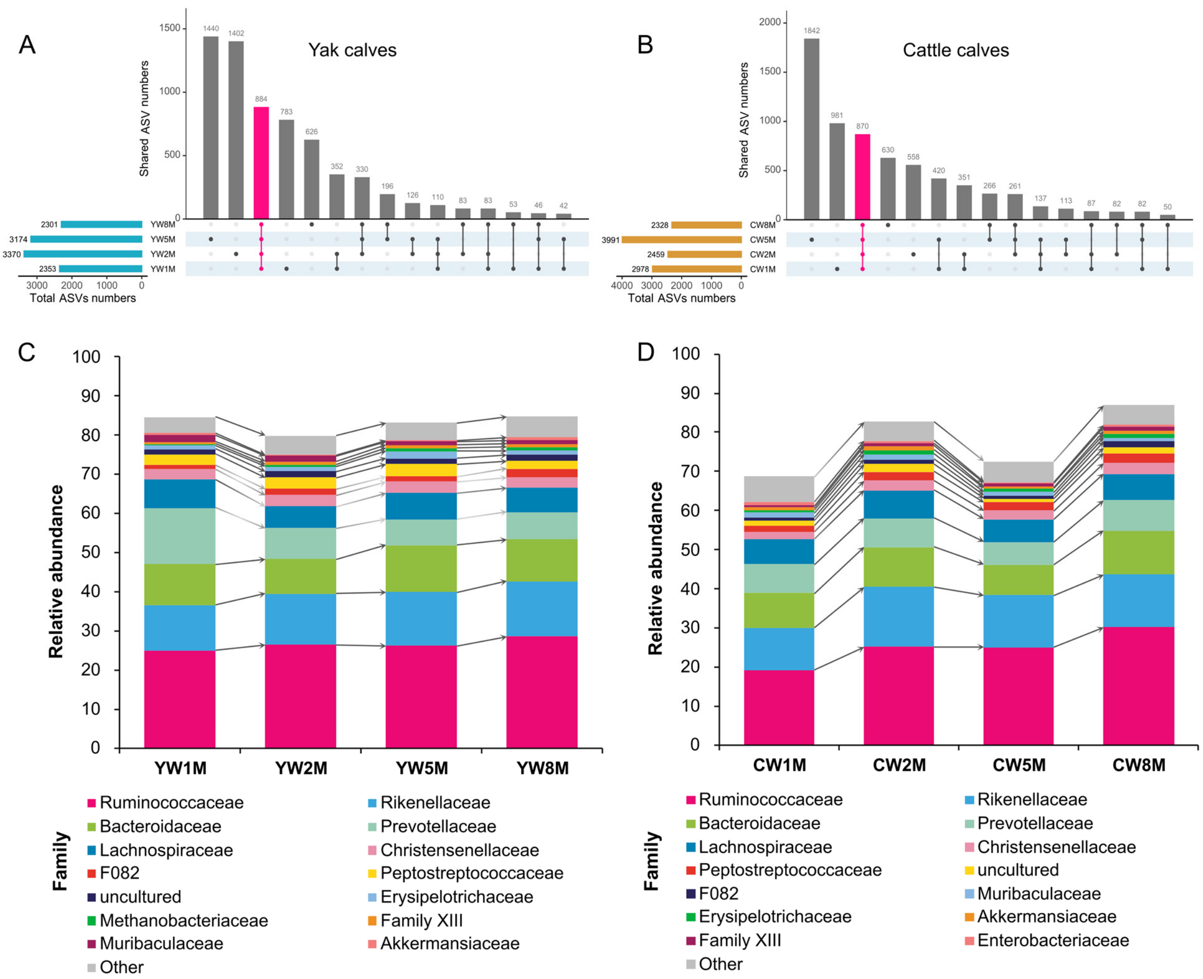

Figure S4. Analysis of the core fecal microbial community in yak and cattle calves after weaning. UpSet plots based on shared ASVs in yak (**A**) and cattle (**B**) calves. Bar plot of taxa relative abundance at family level (top 15) based on shared ASVs in yak (**C**) and cattle (**D**) calves in different months after weaning.
